# Comparative transcriptome analysis of two pomelo accessions with different parthenocarpic ability provides insight into the molecular mechanisms of parthenocarpy in pomelo (*Citrus grandis*)

**DOI:** 10.3389/fpls.2024.1432166

**Published:** 2024-07-29

**Authors:** Keke Zhao, Yunchun Zhang, Sulei She, Ziwei Yang, Yue Zhang, Weiping Nie, Xu Wei, Haiyan Sun, Jiangbo Dang, Shuming Wang, Di Wu, Qiao He, Qigao Guo, Guolu Liang, Suqiong Xiang

**Affiliations:** ^1^ Key Laboratory of Agricultural Biosafety and Green Production of Upper Yangtze River (Ministry of Education), College of Horticulture and Landscape Architecture, Southwest University, Chongqing, China; ^2^ College of Horticulture, State Key Laboratory of Crop Genetics and Germplasm Enhancement and Utilization, Nanjing Agricultural University, Nanjing, China; ^3^ Citrus Research and Education Center, University of Florida, Lake Alfred, FL, United States

**Keywords:** parthenocarpy, pomelo, cell division, IAA, transcriptome

## Abstract

Parthenocarpy is an important way for seedless fruit production in citrus. However, the molecular mechanism(s) of parthenocarpy in pomelo is still unknown. Our initial study found significantly different parthenocarpic abilities in Guanximiyou (G) and Shatianyou (S) pomelo following emasculation, and an endogenous hormone content assay revealed that indole-3-acetic acid (IAA), gibberellic acid (GA_3_) and zeatin (ZT) jointly promoted fruit expansion and cell division in parthenocarpic pomelo (G pomelo). To unravel the underlying molecular mechanism(s), we conducted the first transcriptome analysis on the two pomelo accessions at these two critical stages: the fruit initiation stage and the rapid expansion stage, in order to identify genes associated with parthenocarpy. This analysis yielded approximately 7.86 Gb of high-quality reads, and the subsequent *de novo* assembly resulted in the identification of 5,792 DEGs (Differentially Expressed Genes). Among these, a range of transcription factor families such as *CgERF*, *CgC2H2*, *CgbHLH*, *CgNAC* and *CgMYB*, along with genes like *CgLAX2*, *CgGH3.6* and *CgGH3*, emerged as potential candidates contributing to pomelo parthenocarpy, as confirmed by qRT-PCR analysis. The present study provides comprehensive transcriptomic profiles of both parthenocarpic and non-parthenocarpic pomelos, reveals several metabolic pathways linked to parthenocarpy, and highlights the significant role of plant hormones in its regulation. These findings deepen our understanding of the molecular mechanisms underlying parthenocarpy in pomelo.

## 1 Introduction

Parthenocarpy refers to the phenomenon of producing seedless fruits either without pollination or pollination but without fertilization. Parthenocarpy can be categorized into natural parthenocarpy (autonomous parthenocarpy) and induced parthenocarpy, depending on whether external stimulation is required. Natural parthenocarpy further includes obligatory parthenocarpy and facultative parthenocarpy. In sexually propagated species, parthenocarpic genotypes are often facultative due to lower trait expressivity. Conversely, vegetatively propagated crops often adopt obligate parthenocarpy (Gorguet et al., 2005). Regardless of the type of parthenocarpy, seedless fruit will eventually be produced (Zhao et al., 2021). Parthenocarpy plays a crucial role as an economically and agronomically important trait in fruit trees by reducing the dependence on pollination for fruit and vegetable production.

Seedless fruits are highly favored for their superior quality and ease of processing, and especially in the citrus market, seedless or low-seeded varieties hold strong competitive advantages (Pandolfini, 2009; Zhou et al., 2018). Parthenocarpy significantly influences the yield of seedless citrus, thereby impacting cultivation practices and management costs (Olimpieri et al., 2007; Lietzow et al., 2016). Parthenocarpy in citrus is characterized by both obligatory and facultative types. Obligatory parthenocarpic citrus varieties like Satsuma mandarin and navel orange exhibit strong parthenocarpic ability and fruit setting rates (Talon et al., 1992). However, the fruit setting rate of facultative parthenocarpy types, such as Clementine tangerine, is relatively lower (Mesejo et al., 2013). Certain self-incompatible pomelo species also possess a degree of parthenocarpic ability (Iwamasa and Oba, 1980; Hoang et al., 2014).

Studies on parthenocarpy in citrus have shown that the variation in parthenocarpic ability is related to the content of endogenous hormones, particularly gibberellin and auxin, in different germplasm (Talon et al., 1992; Mesejo et al., 2016). Mesejo et al. (2013) demonstrated that ‘Marisol’ exhibits a higher GA_1_ in the ovary compared to ‘Clemenules’, aligning with its higher parthenocarpic ability. Studies have demonstrated that the level of GA_3_ increased in both pollinated and unpollinated ‘W-murcott’ tangor whole fruits, with a higher GA_3_ content detected in unpollinated fruits compared to pollinated ones between 25-50 days after anthesis (Dong et al., 2020), and the higher level of GA_3_ equivalents promoted the development of parthenocarpy (Talon et al., 1990). Therefore, active GA_1_ and GA_3_ promote the development of parthenocarpic citrus generally at the young fruit stage. Moreover, the high expression of GA20ox and GA3ox genes, involved in gibberellin synthesis, is speculated to play a role in promoting the synthesis of active gibberellins and facilitating parthenocarpy in citrus (Vriezen et al., 2008; Mesejo et al., 2013).

Many other studies have shown that higher auxin levels during the bud stage of citrus parthenocarpic materials promote fruit setting and development. Comparatively, in parthenocarpic seedless germplasm, the ovary of Satsuma mandarin exhibits significantly higher auxin concentration during the bud stage than navel oranges (‘Robertson’ and ‘Washington’ navel orange) and two seedless varieties of Valencia (‘Armstrong’ and ‘Rico No.1’) (Gustafson, 1939), suggesting a stronger parthenocarpic ability in Satsuma mandarin. Gustafson’s research (Gustafson, 1939) found that the IAA content in Satsuma mandarin remains consistently low within the first four days after flowering, indicating that IAA has minimal influence on the development of post-flowering young fruits in parthenocarpic citrus varieties (Zhang et al., 1994). Xiao et al. (2005) observed lower IAA content in the pericarp and flesh of parthenocarpic citrus compared to self-flowering citrus during the fruit-growing stage, suggesting a reduced auxin requirement for parthenocarpic fruit development. However, Dong et al. (2020) reported an upward trend in IAA content in unpollinated ‘W-murcott’ tangor fruits at 39 days after anthesis, with higher levels than in pollinated fruits, promoting parthenocarpy in ‘W-murcott’ tangor. Overall, it is confirmed that parthenocarpic citrus exhibits higher ovary auxin content during early anthesis, with no significant impact from the overall decline after full anthesis on fruit setting. Moreover, studies have shown that Aux/IAA and ARF genes are involved in parthenocarpy regulation, although most of these studies have primarily focused on herbaceous plants such as tomatoes and strawberries. Research on citrus is currently at the stage of uncovering the key genes involved in parthenocarpy. In a study by Liao et al. (2017), it was observed that the mRNA expression levels of post-flower auxin-related genes *CmsIAA9*, *AUCSIA*, and *PIN4* in *Citrus medica* L. var. *sarcodactylis* Swingle were down-regulated. Particularly, the gene *CmsIAA_9_* in the pistil base showed significant down-regulation compared to citron at 3 days after anthesis, indicating a potential link between low *CmsIAA9* transcription and parthenocarpy.

Cytokinin, an adenine derivative of N-6, is important for early fruit setting and development. However, research specifically focused on cytokinin-related parthenocarpy is limited. Zeatin nucleoside (ZT), a natural cytokinin, was found to be higher in Satsuma mandarin during early fruit development, promoting overall fruit growth, and specifically stimulated rapid peel growth during the fruit growth stage (Xiao et al., 2005). Cytokinin, along with gibberellin and auxin, is believed to contribute to the development of parthenocarpic young fruit, with cytokinin playing a significant role in fruit enlargement in citrus. Studies on other growth inhibiting hormones, such as abscisic acid and ethylene, in parthenocarpic citrus varieties are still relatively few. Generally, the ratio of growth promoting hormones to inhibiting hormones is used to reflect the synergistic effect between hormones.

‘*Citrus grandis* Osbeck.cv. Shatianyou’ (S pomelo) and ‘*Citrus grandis* Osbeck.cv. Guanximiyou’ (G pomelo) are very important pomelo varieties in China. S pomelo, originated in Shatian, Rongxian, Guangxi Province, is mainly cultivated in Guangxi, Guangdong, Chongqing and other places. G Pomelo, originated in Pinghe County, Fujian Province, is cultivated in Fujian and introduced to other pomelo producing provinces. Both are self-incompatible. The G pomelo is known for its ability to produce seedless fruits when cultivated on a large scale (Deng, 2008) On the other hand, the S pomelo has a naturally low fruit setting rate, often necessitating artificial pollination to achieve high yield. While pollination improves the fruit setting rate, it leads to the development of numerous seeds (Zhao et al., 2019) Therefore, developing seedless pomelo cultivars is a primary breeding objective of S pomelo. However, research on parthenocarpic pomelo is limited. In this study, we analyzed the reproductive and developmental characteristics of S pomelo and G pomelo, investigated the dynamic changes of endogenous hormones, and performed transcriptomic sequencing (RNA-seq) at specific stages. Our aim was to identify candidate genes associated with parthenocarpy, thereby establishing an important foundation for understanding the molecular mechanisms of parthenocarpy in pomelo.

## 2 Materials and methods

### 2.1 Plant materials and experimental design

The study utilized adult pomelos from two accessions, namely S pomelo and G pomelo, with each accession consisting of 5 equally vigorous plants. S pomelo has a low tendency to develop parthenocarpic fruits, while G pomelo has a high tendency. The plants were cultivated using standard practices at Shouwan Orchard, Changshou District, Chongqing, China. Before anthesis (referred to as 0 days after anthesis), flower buds from each tree were randomly selected from four directions for different treatments including cross-pollination (S♀×G♂ and G♀×S♂), artificial self-pollination (S♀×S♂ and G♀×G♂), and emasculation (SE and GE). All treated combinations were immediately bagged to prevent foreign pollen. Random samples were taken from each tree in four directions, and the sampling materials were not included in the fruit setting statistics. Some ovaries and styles were immediately fixed in formalin–acetic acid–alcohol (FAA) solution at room temperature for ovary paraffin sectioning and style aniline blue dyeing (Martin, 1959; Dries, 2008), while the remaining ovaries were stored at -80°C for RNA extraction.

### 2.2 Evaluation of parthenocarpic ability

Ten fruitlets were collected from each treatment at -5, 0, 5, 10, 16, 20, and 26 days after anthesis (DAA) to measure fruitlet fresh weight, vertical and transverse diameters, and compare the growth trends of each treatment. The fruit setting rates were investigated three times at 1, 2 and 4 months after anthesis (MAA), and the treated fruits were harvested in December. Fruit number per treatment and seed number per fruit were counted, and their fruit weight was measured to compare the difference between the seedless and seeded fruits in the same accession. The ratio of weight of the emasculated seedless fruits to that of the cross-pollinated seeded fruit was used as parthenocarpic fruit weight rate. The parthenocarpic degree in each accession was evaluated as a combination value of the seedless fruit setting rate and parthenocarpic fruit weight rate, i.e., the sum of the seedless fruit setting rate (%) and parthenocarpic fruit weight rate (%) divided by 2 (Zhou et al., 2018).

### 2.3 Phytohormone measurements

For hormone analyses (IAA, ZT, GA_3_ and ABA), freeze-dried ovary tissues (about 200 mg dry weight) ground into fine powder in the presence of liquid nitrogen. The powdered tissues were extracted with 80% methanol (De Vos et al., 2007). The extracting mixture was centrifuged at 12,000 rpm for 10 min, and the supernatant was collected. Subsequently, the precipitation secondary extraction protocol described by Zhang et al, was used with some modifications, and the pH was adjusted to 3.0 with hydrochloric acid (Zhang et al., 2017). The combined supernatant was consecutively extracted with petroleum ether, ethyl acetate, and then evaporated to dryness at 40°C. Finally, the mobile phase solution was added and dissolved by vortex vibration to create the analytical solution, which was filtered through a 0.22 μm filter membrane. The hormones were separated using an autosampler and C18 (150 mm×4.6 mm, 5 μm) reversed phase column. The mobile phase was composed of 100% methanol (phase A) and 0.1% glacial acetic acid buffer (phase B), with a mobile phase ratio of 55% (A) to 45% (B), and a flow rate of 0.8 mL/min. The detection wavelength was set at 254 nm, and the injection volume was 20 μL.

Hormone content was quantified by measuring the peak area and referring to a standard curve. The normalized treatment for each hormone content was denoted by *K*, and the hormone balance was measured by T value [(*K_IAA_* +*K_GA3_* +*K_ZT_*)/*K_ABA_*] (Dong et al., 2020).

### 2.4 Analysis of quality characteristics in ripe pomelos under different treatments

From each treatment, five ripe fruits were selected and processed to extract their juice. The juice was then strained through a fine gauze to eliminate any remaining solids. Using a pipette, the juice was carefully applied to the sample slot of a portable refractometer to determine the concentration of soluble solids. This measurement was repeated three times.

For the citric acid analysis, the samples were ground into powder in liquid nitrogen, and approximately 0.1 to 0.2 grams were weighed and dissolved in 1.5 mL ultrapure water, followed by ultrasonication for 15 minutes. The mixture was then centrifuged at 12,000 rpm for 10 minutes, and the supernatant was extracted and filtered through a 0.22 μm filter head for analysis. The high-performance liquid chromatography (HPLC) was set up with the following parameters: a Shimadzu SPD detector; a mobile phase consisting of a solution of 0.02 mol/L potassium dihydrogen phosphate (2.772 g dissolved in 1 L ultrapure water and adjusting pH to 2.52 with phosphoric acid) mixed with methanol in a 90:10 ratio; a C18 column; a flow rate of 0.8 mL/min; a column temperature of 30°C; a detection wavelength of 210 nm; and an injection volume of 10 μL.

The extraction method for glucose, fructose, and sucrose followed the same protocol as for citric acid. The HPLC conditions for these sugars were slightly different: a Shimadzu RID detector; a mobile phase of acetonitrile and water in a 7:3 ratio; an amino column; a flow rate of 0.8 mL/min; a column temperature of 40°C (with the RID cell temperature set at 40°C); injection volume of 10 μL. Calibration was achieved using the following standard curves:


$$\text{Citric acid}:\text{ Y }=\;913505\text{x }+\;24895;\;{\text{R}}^{2}\;=\;0.9997;$$

$$\text{Glucose}:\text{ Y }=\;100566\text{x }-\;762.17;\;{\text{R}}^{2}\;=\;0.9999;$$

$$\text{Fructose}:\text{ Y }=\;100772\text{x }-\;21678;\;{\text{R}}^{2}\;=\;0.9998;$$

$$\text{Sucrose}:\text{ Y }=\;100540\text{x }+\;24895;\;{\text{R}}^{2}\;=\;0.9997.$$

### 2.5 RNA isolation and transcriptome analysis

Based on the physiological data mentioned above, the critical stages of pomelo fruit development were identified as fruit initiation at 10 DAA and rapid expansion at 26 DAA. The ovaries of SE and GE at 10 DAA and 26 DAA (three replicates each) were used for RNA-seq analysis, namely SE1, SE2, GE1, and GE2. Total RNA was extracted using the RNA prep Pure TIANGEN kit (TIANGEN, DP441, Beijing). The cDNA library and sequencing library were constructed following the manufacturer’s instructions of RNA Kit for Sequencing (APExBIO, Cat. No. K1159, America) and Tn5 DNA Library Prep Kit for Illumina (APExBIO, Cat. No. K1055, America).

The raw sequencing data was processed using the software Fastp (https://github.com/OpenGene/fastp) to remove reads with >10% unknown bases and those of low quality. The filtered reads were then mapped to the reference sequences using Hisat2 (version2.1.0) (Kim et al., 2013), allowing for a maximum of two base mismatches in the alignment. The gene expression level was calculated by Transcripts Per Kilobase Million (TPM). The DESeq2 algorithm was utilized to identify the differentially expressed genes between different transcriptomes (Anders and Huber, 2010). A threshold of False Discovery Rate (FDR)< 0.01, p-value< 0.05, and an absolute value of log2ratio > 1 was used to determine the significance of differential gene expression.

Gene Ontology (GO) enrichment analysis was performed to identify significantly enriched GO terms in the DEGs compared to the genome background. Statistical enrichment of DEGs in Kyoto Encyclopedia of Genes and Genomes (KEGG) pathways was tested using KOBAS software (Mao et al., 2005). GO terms showing a corrected P-value ≤ 0.05 were considered to be significantly enriched. The top 20 pathways with the smallest P-value were analyzed to determine the DEGs content in KEGG pathways.

### 2.6 RNA-seq validation and spatio-temporal expression quantification by qRT-PCR

Nine genes were randomly selected for RNA-seq data validation using qRT-PCR. The primers used for qRT-PCR are shown in **Supplementary Table S1**. Total RNA was extracted from the ovaries of SE1, SE2, GE1, and GE2 using RNA prep Pure Plant Kit (TIANGEN). Reverse transcription was performed on 1 μg aliquots of total RNA using the Prime Script RT Kit (Takara, Dalian, China) following the manufacturer’s instructions. In addition, the spatio-temporal expression of hormone-related genes was quantified by extracting total RNA and performing reverse transcription from ovaries at various stages of cross-pollination and emasculation. The qRT-PCR with three replicates was performed by using the SYBR Premix Ex Taq™ Kit (TAKARA) following the description in the handbook of the Bio-Rad iQ1 real-time PCR system (Bio-Rad). The CT values of internal reference gene *β-tubulin* of Guanximiyou and target genes were read and calculated using the 2^-ΔΔCT^ formula.

## 3 Results

### 3.1 S pomelo and G pomelo exhibit differences in their morphology and cell division

**Figure 1:**
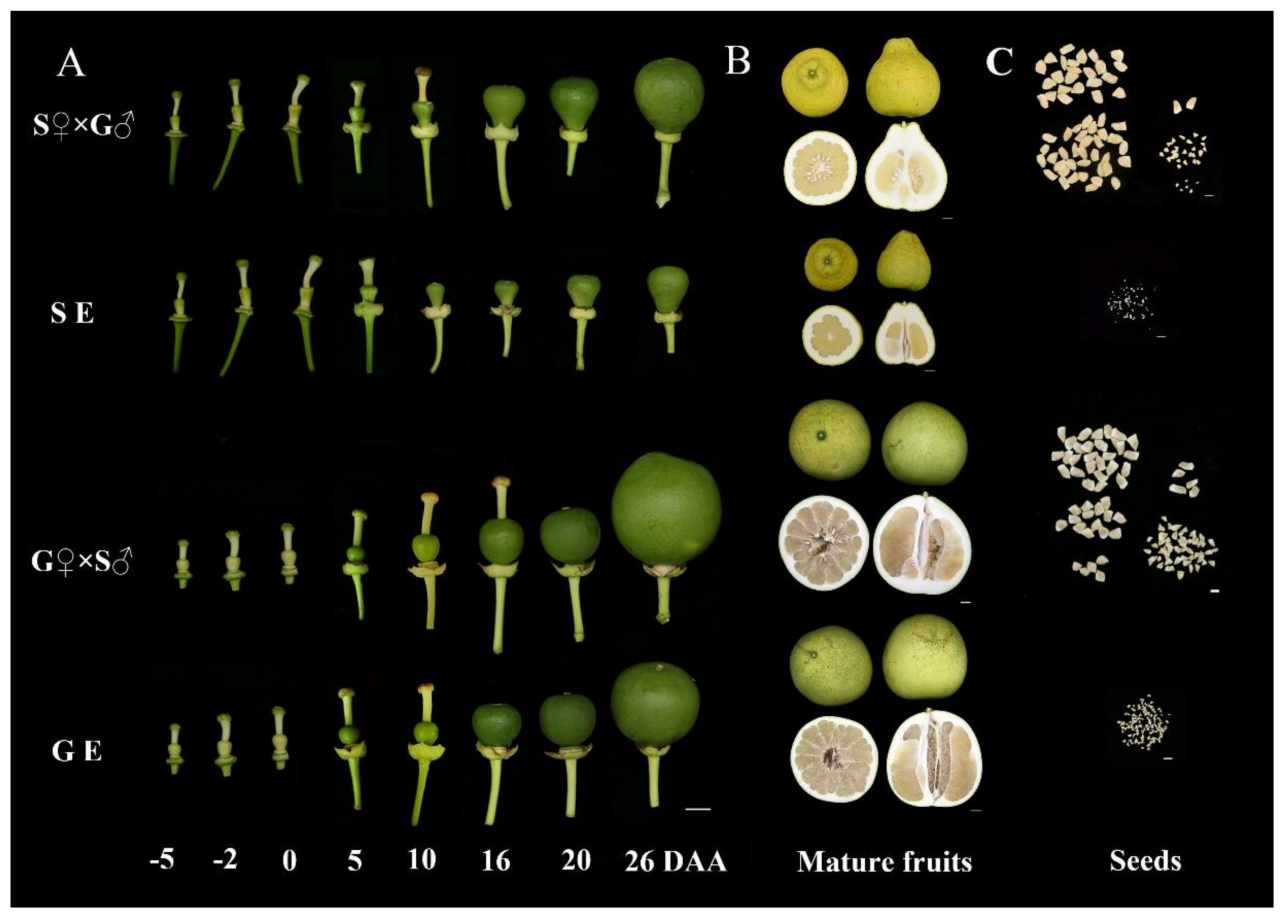
Morphological changes of pomelo fruit and seed during different stage. **(A)** Observation of the effect of cross-pollination (S♀×G♂ and G♀×S♂) and emasculation treatment (SE and GE) of S pomelo and G pomelo on young fruit development. DAA, days after anthesis. Scale bar: 1 mm. **(B)** Morphological observation of mature fruits in each treatment. Scale bar: 2 cm. **(C)** Morphological observation of seeds in each treatment. Scale bar: 1 cm.

To investigate the effect of emasculation on ovary development and parthenocarpy process, we compared the fruitlet morphology of emasculation-treated ‘S pomelo’ and ‘G pomelo’ cultivars with cross pollination-treated at different time points (**Figure 1A**). The size of fruitlet in each treatment at different time points is shown in **Supplementary Figure S1**. The fresh weight, vertical and transverse diameters of ovary in each treatment showed a gradual increasing trend. The ovary began to obviously grow at 10 DAA, and the fruitlet expanded rapidly at 26 DAA in both cultivars. Compared to the previous time point, the ovary weight of emasculated S pomelo (SE) increased by 12.72% at 10 DAA and 109.5% at 26 DAA. While, the ovary weight of emasculated G pomelo (GE) increased by 107.4% and 170.2%, respectively (**Supplementary Figure S1A**). The vertical and transverse diameters ovary in both cultivars showed similar patterns to ovary weights at these two critical stages, implying a significantly higher parthenocarpic growth rate in G pomelo compared to S pomelo (**Supplementary Figures S1A, B**). In addition, the fruit of S Pomelo is pear shaped, but the fruit of G pomelo is spherical (**Figure 1B**). The fruit size of G pomelo is significantly larger than that of S pomelo, but the peel thickness of S pomelo is significantly higher than that of G pomelo (**Figure 1B**). Emasculation can significantly reduce the number of S and G pomelo full seeds (**Figure 1C**).

**Figure 2:**
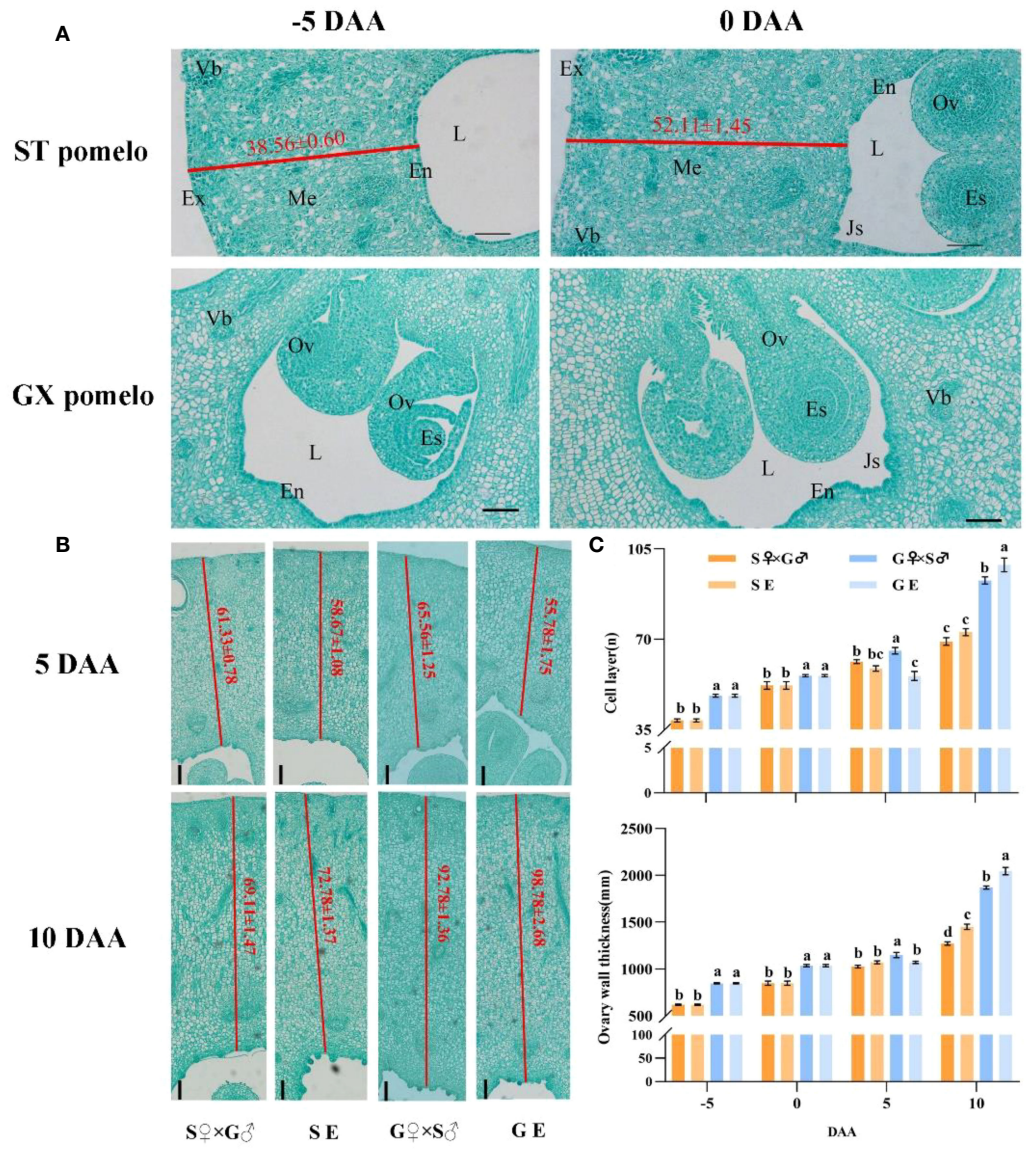
Time-course of cell growth from the ovary wall of S pomelo and G pomelo. **(A)** Cross-sction of the pomelo ovary at -5 and 0 DAA. En, Endocarp; Ex, Exocarp; Me, Mesocarp; Vb, Vascular bundle; Ov, Ovule; Es, Embryo sac; L, Locule; Js, Juice sacs. The values in red color indicate the numbers of the cell layers from the exocarp to the endocarp. **(B)** Ovary cell division at 5 and 10 days after cross-pollination (S♀×G♂ and G♀×S♂) and emasculation treatment (SE and GE). Scale bar: 0.1 mm. **(C)** Time-course analysis of the number of cell layers and ovary wall thickness. Values are means ± standard errors (SEs) of three ovaries. Different letters denote significant differences according to Duncan’s range test at *p*< 0.05.

Cytological observation showed that the growth of pomelo fruit was initiated by an increase in cell numbers in the pericarp and ovule tissues, leading to the rapid filling of the locules among the endocarps with newly divided cells (**Figure 2A**). During anthesis, clear juice sacs appeared in the endocarp, and these juice sacs continue to develop until they completely filled the locules at fruit maturity (**Figure 2A**). The thickness of the ovary wall increased with the accumulation of cell layers. However, from -5 to 5 DAA, pericarp cells division was slow, but exhibited significant development at 10 DAA (**Figures 2B, C**). At 10 DAA, there was no significant difference in the number of cell layers between S pomelo cross-pollination (S♀×G♂) and emasculation treatment (SE), while the number of cell layers in the G pomelo emasculation treatment (GE) were significantly higher than that in the cross-pollination treatment (G♀×S♂) (**Figures 2B, C**). Moreover, at 10 DAA, the number of cell layers in emasculated G pomelo (GE) increased by 77.09% compared to 5 DAA, while in emasculated S pomelo (SE), it only increased by 24.05%.

### 3.2 Fruit setting rate and parthenocarpic rate

At 1 month, 2 months and 4 months after anthesis (MAA), the fruit setting rates of emasculation were lower compared to their respective cross-pollination. The fruit setting rates of emasculated G pomelo were consistently higher than those of emasculated S pomelo (**Table 1**). The parthenocarpic rates were calculated by combining the fruit setting rates of seedless fruits with the parthenocarpic fruit weight rates. The results showed that the parthenocarpic rate of G pomelo was 50.77%, while that of S pomelo was 29.75% (**Supplementary Table S2**, **Table 1**). This indicates that the parthenocarpic rate of G pomelo was significantly higher than that of S pomelo.

**Table 1:** Fruit setting rate and parthenocarpic rate of seedless fruit in each treatment.

Treatment	Number of treatment	1 MAA		2 MAA		4 MAA		Parthenocarpic rate/%
		Number of fruit set	Fruit setting rate/%	Number of fruit set	Fruit setting rate/%	Number of fruit set	Fruit setting rate/%	
S♀×G♂	302	86	28.48%	64	21.19%	61	20.20%	/
S E	514	56	10.89%	12	2.33%	10	1.95%	29.75 ± 22.65%
G♀×S♂	246	56	22.76%	37	15.04%	32	13.01%	/
G E	221	43	19.46%	15	6.79%	12	5.43%	50.77 ± 5.17%*

The asterisk indicates a significant difference at the 0.05 level according to T-test. The symbol “/” was used to denote 'not applicable' or 'no data available' for a particular entry.

### 3.3 Dynamic changes of hormone levels during early fruit development

Since phytohormones have been found to play an important role in promoting parthenocarpy during early fruit development, we measured the phytohormone levels over this time course.

**Figure 3:**
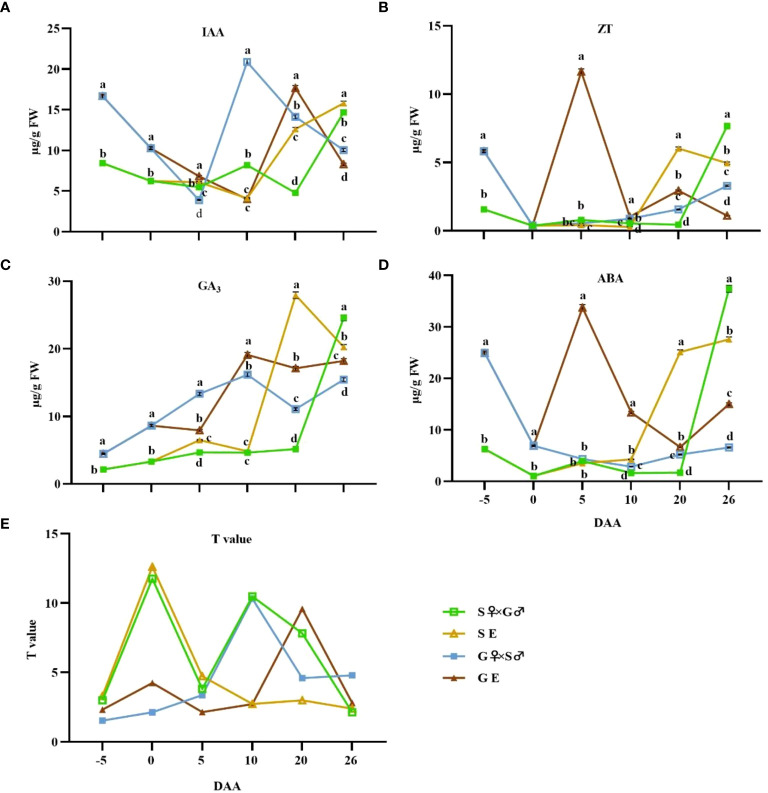
Dynamic changes of endogenous hormone contents and T values during early fruit development at -5, 0, 5, 10, 20, and 26 DAA after cross pollination and emasculation. **(A)** Indole-3-acetic acid (IAA) content. **(B)** Zeatin (ZT) content. **(C)** Gibberellic acid (GA_3_) content; **(D)** ABA content. **(E)** T value. Values are mean ± SEs of 3 biological replicates. Different letters denote significant differences according to Duncan’s multiple range test at *p*< 0.05.

From -5 to 26 DAA, the IAA level in the ovaries of G pomelo exhibited a pattern of initial decrease, followed by an increase, and then another decrease, whereas the IAA content in S pomelo fruit initially decreased and then elevated (**Figure 3A**). The bud stage of pomelo ovaries accumulated higher levels of IAA, and the IAA content in the ovaries of G pomelo was significantly higher than that in the S pomelo at -5~0 DAA (**Figure 3A**). Although the peak times differed between the treatments, the peak values of pollination and emasculation treatments of G pomelo were higher compared to the corresponding treatments in S pomelo.

The GA_3_ content of G pomelo under pollination and emasculation treatments showed an overall increasing trend at -5~10 DAA, followed by slight fluctuation (**Figure 3C**). However, from 10~26 DAA, the GA_3_ levels of G pomelo after emasculation were significantly higher than those in the corresponding pollination treatments (**Figure 3C**). In addition, the GA_3_ levels of G pomelo fruits were remarkably higher than those of S pomelo fruits at -5~10 DAA (**Figure 3C**).

The levels of ZT in the ovaries of pollination and emasculation treatments of S pomelo and pollination treatment of G pomelo were consistently low from 0 to 10 DAA, with their contents not exceeding 1 μg/g (FW). However, they began to increase at 10 DAA (**Figure 3B**). In contrast, the emasculated G pomelo reached a peak value of 11.64 μg/g (FW) at 5 DAA (**Figure 3B**).

The variation trend of ABA content in G pomelo treated by emasculation was similar to that of ZT, reaching a peak value of 33.73 μg/g (FW) at 5 DAA (**Figure 3D**). However, fruit development is synergistically regulated by multiple hormones, and this synergy is usually represented by the ratio of promoting to inhibitory hormones. In this study, we used T value to characterize the synergistic effect of phytohormones (**Figure 3E**). The T value of S pomelo at -5~5 DAA was higher than that of G pomelo, and both pollination and emasculation of S pomelo reached their peak at 5 DAA, suggesting that the S pomelo ovaries accumulated a lot of growth-promoting hormones during the flowering period, thereby promoting fruitlets growth (**Figure 3E**). The T value of the ovary increased sharply in S pomelo hybridization and emasculation treatments at 5 DAA and in G pomelo emasculation treatments at 10 DAA. However, the T value of S pomelo emasculation treatments did not increase (**Figure 3E**).

### 3.4 The fruit quality of G pomelos is not affected by emasculation treatment

Statistical analysis was conducted on at least five mature fruits from each treatment. The weight of individual fruits and the rate of parthenocarpic fruit weight for the G pomelo were significantly greater than that for the S pomelo (**Supplementary Table S2**). It is worth noting that the weight of the parthenocarpic fruit from S pomelo after emasculation was approximately 518 g, significantly lower than that of natural fruits and hybrid S pomelo fruits. In contrast, the weight of the parthenocarpic fruit from G pomelo after emasculation was approximately 1572 g, with no significant difference from natural fruits and hybrid G pomelo fruits. This indicates that parthenocarpic fruits of G pomelo can reach an ideal size under natural conditions, while those of S pomelo cannot. The number of plump seeds in the emasculated fruits of both varieties was zero (**Supplementary Table S2**), indicating seed abortion but with the capability for parthenocarpy.

To understand whether the quality of the parthenocarpic fruits has changed after emasculation, the soluble solids, citric acid, glucose, fructose, and sucrose content of mature fruits from different treatments were comparatively analyzed (**Table 2**). The results showed that the soluble solids content in all treatments of G pomelo was significantly higher than that of the S pomelo, and emasculation did not have a significant impact on the soluble solids of both pomelo types. The content of citric acid and sucrose in the emasculated fruits of S and G pomelos did not differ significantly from other treatments within each variety. However, the content of glucose and fructose in the SE fruits was significantly lower than that in the hybrid treatment of S pomelo, while the glucose content in GE fruits was not significantly different from its hybrid treatment. In summary, GE treatment had significantly higher contents of soluble solids, citric acid, and glucose compared to SE, and also had higher contents of fructose and sucrose than SE pomelo.

**Table 2:** Analysis of fruit quality in each treatment.

Treatment	Soluble solids/%	Citric acid/mg·g^-1^	Glucose/mg·g^-1^	Fructose/mg·g^-1^	Sucrose/mg·g^-1^
S natural	9.616 ± 0.21b	2.09 ± 0.06c	5.98 ± 0.40c	8.94 ± 0.89c	53.51 ± 3.69
S♀×G♂	9.508 ± 0.29b	2.39 ± 0.11c	13.08 ± 1.09a	17.00 ± 0.78a	75.04 ± 7.21
S E	9.9 ± 0.27b	2.56 ± 0.27bc	5.96 ± 0.51c	9.85 ± 0.56c	61.72 ± 5.02
G natural	11.506 ± 0.39a	4.03 ± 0.66ab	8.65 ± 0.63bc	12.35 ± 0.38bc	57.57 ± 3.13
G♀×S♂	11.578 ± 0.18a	4.45 ± 0.12a	10.73 ± 0.66ab	14.07 ± 1.32ab	61.48 ± 4.73
G E	11.814 ± 0.48a	4.64 ± 0.29a	10.14 ± 0.34ab	10.17 ± 0.25c	64.40 ± 2.30

Different letters indicate a significant difference of p< 0.05 according to Tukey’s Honest Significant Difference (HSD) test.

### 3.5 An overview of pomelo fruit transcriptome

In order to understand the molecular differences underlying the contrasting parthenocarpic ability of S pomelo (low) and G pomelo (high), we performed transcriptome sequencing on the two cultivars during two critical fruit development stages (10 DAA and 26 DAA). The RNA sequencing obtained over 7.86 Gb of data per sample, with Q20 values and Q30 values exceeding 98.10% and 94.60%, respectively. The average GC content of these samples was 51.16% (**Supplementary Table S3**). Clean reads of each sample were aligned to the designated reference genome(https://www.ncbi.nlm.nih.gov/genome/10702?genome_assembly), resulting in alignment efficiencies ranging from 82.09% to 92.90%. These sequences were assembled into 30,113 unigenes (**Supplementary Table S3**). The Pearson correlation coefficients between biological replicates are shown in **Supplementary Figure S2**, with R^2^ -value >0.985.

**Figure 4:**
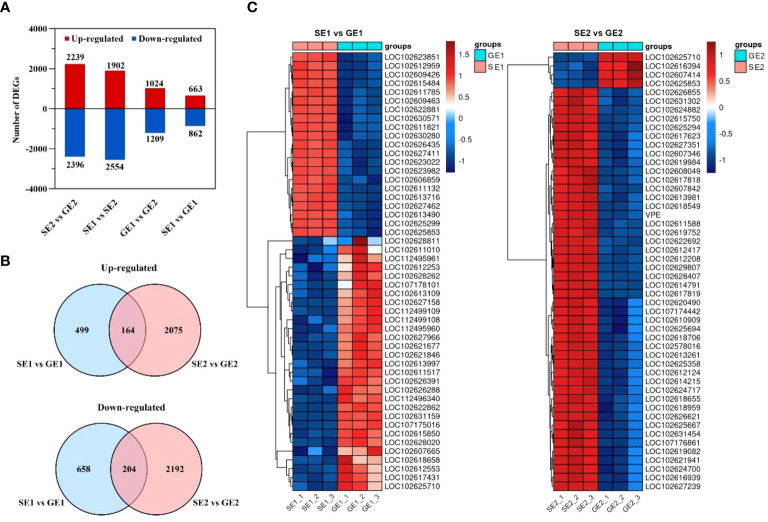
The number of DEGs in SE and GE ovaries at two critical development stage. **(A)** The total number of up-regulated and down-regulated DEGs at four comparison groups; **(B)** Venn diagram of up-regulated genes and venn diagram of down-regulated genes between SE1 vs GE1 and SE2 vs GE2; **(C)** Heatmap diagrams showing the relative expression levels of the top 50 DEGs with the smallest P values for the SE1 vs GE1 comparable group and the SE2 vs GE2 comparable group.

To identify genes potentially involved in parthenocarpy development, we analyzed the differential gene expression in pomelo ovaries. A total of 5,792 DEGs were identified through pairwise comparisons, with 4,635, 4,456, 2,233 and 1,525 DEGs were found between SE2 and GE2, SE1 and SE2, GE1 and GE2, and SE1 and GE1, respectively (**Figure 4A**). Among these, 2,239 genes were up-regulated and 2,396 genes were down-regulated from SE2 vs. GE2. Comparison of SE2 with SE1 revealed 1,902 genes that were up-regulated and 2,554 genes that were down-regulated in the later. 1,024 genes were up-regulated and 1,209 genes were down-regulated from GE2 compared with GE1. Besides, 663 genes were up-regulated and 862 genes were down-regulated from SE1 vs. GE1 (**Figure 4A**). Notably, 164 of the 663 up-regulated DEGs and 204 of the 862 down-regulated DEGs between SE1 vs GE1 were also among the DEGs between SE2 vs. GE2 (**Figure 4B**).

Furthermore, heatmaps were used to visualize the expression pattern, represented by differential gene log2(TPM+1), of the top 50 DEGs with the smallest P values for the SE1 vs. GE1 group and the SE2 vs. GE2 group (**Figure 4C**). The red and blue points in the heatmaps represented high and low TPM expression levels of the gene, respectively. And genes with similar expression patterns may have common functions or be involved in common metabolic pathways and signaling pathways. The heatmaps showed that GE had 42% of the down-regulated DEGs in the first stage compared to SE, while 92% of the down-regulated DEGs were observed in the second stage (**Figure 4C**).

### 3.6 Functional classification of the DEGs by GO and KEGG pathway analysis

**Figure 5:**
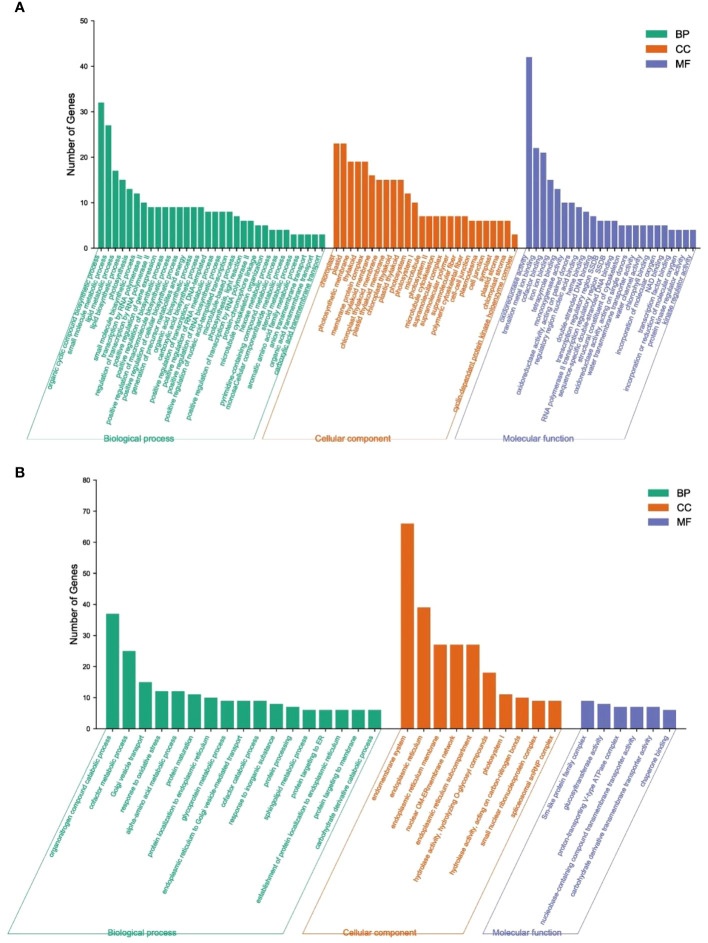
GO analysis of DEGs between SE and GE. **(A)** SE1 vs GE1; **(B)** SE2 vs GE2; BP, biological process; CC, cellular component; MF, molecular function.

To facilitate the global analysis of gene expression, the DEGs between emasculated S pomelo and emasculated G pomelo in the two periods (SE1 vs GE1 and SE2 vs GE2, respectively) were subjected to GO enrichment analysis using the GOseq R package. Three main GO categories were enriched in the DEGs: “biological process” (BF), “cellular component” (CC) and “molecular function” (MF) (**Figure 5**). In the biological process category, the GO terms significantly enriched in the SE1 vs. GE1 comparison included “organic cyclic compound biosynthetic process”, “small molecule metabolic process” and “lipid metabolic process”. The GO terms significantly enriched in the SE2 vs. GE2 comparison included “organonitrogen compound catabolic process”, “cofactor metabolic process” and “Golgi vesicle transport”. In the cellular component category, “chloroplast”, “plastid” and “photosynthetic membrane” were significantly enriched in the SE1 vs. GE1 comparison. “Endomembrane system”, “endoplasmic reticulum” and “endoplasmic reticulum membrane” were significantly enriched in the SE2 vs. GE2 comparison. In the molecular function category, “oxidoreductase activity”, “transition metal ion binding” and “cofactor binding” were significantly enriched in the SE1 vs. GE1 comparison. “Sm-like protein family complex”, “glucosyltransferase activity” and “proton-transporting V-type ATPase complex” were significantly enriched in the SE2 vs. GE2 comparison.

**Figure 6:**
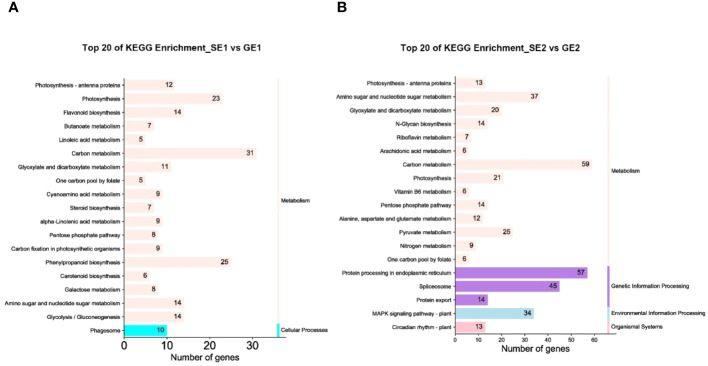
DEGs of the top 20 KEGG pathways with the smallest P value in the SE1 vs GE1 group **(A)** and SE2 vs GE2 group **(B)**.

To further explore the key biochemical and signal transduction pathways involving the DEGs, we performed a pathway enrichment analysis using the KEGG database. As shown in **Figure 6A**, in the SE1 vs. GE1 group, a total of 227 DEGs were significantly enriched in two major metabolic pathways: “Metabolism” (with 217 members) and “Cellular processes” (with 10 members). As shown in **Figure 6B**, in the SE2 vs. GE2 group, a total of 412 DEGs were significantly enriched in four major metabolic pathways: “Metabolism” (with 249 members), “Genetic information processing” (with 116 members), “Environmental information processing” (with 34 members) and “Organismal systems” (with 13 members).

### 3.7 Analysis of transcription factor gene family involved in parthenocarpy of pomelo

**Figure 7:**
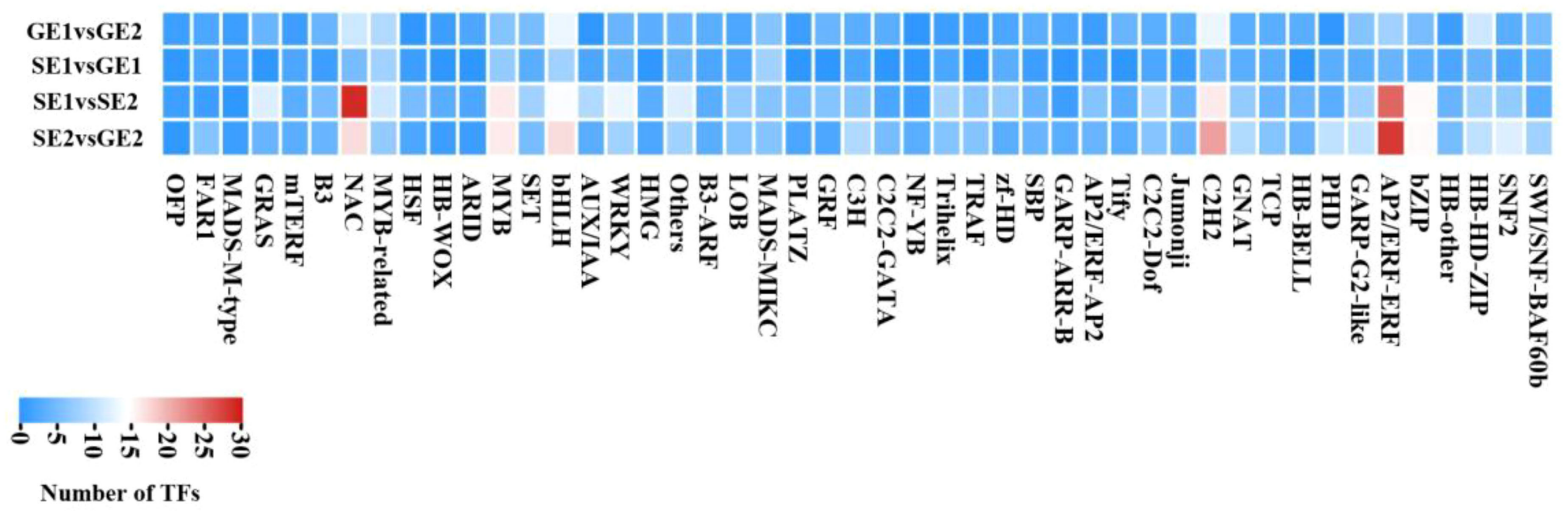
Heatmap of TF families in four comparisons.

To investigate the potential role of transcription factors in pomelo parthenocarpy, we conducted an analysis of differentially expressed transcription factor genes between emasculated S pomelo and emasculated G pomelo during two critical periods (SE1 vs. GE1 and SE2 vs. GE2). The analysis revealed a total of 107 and 318 differentially expressed transcription factors for SE1 vs. GE1 and SE2 vs. GE2, respectively (**Figure 7**). Among the differentially expressed transcription factor (TF) families, *ERF*, *C2H2*, *bHLH*, *NAC* and *MYB* families showed notable representation in both SE2 vs. GE2 and SE1 vs. SE2 comparisons (**Figure 7**). The *ERF* family had the highest number of DEGs (28 and 25), followed by the *NAC* family (17 and 29) (**Figure 7**). In the comparison of SE1 vs. GE1, the *bHLH*- and *MYB*-related classes account for the largest proportion of DEGs (**Figure 7**). Furthermore, the heatmaps showed that the transcription levels of these TFs were altered in the ovaries of both S pomelo and G pomelo after emasculation, with the exception of the MADS box family.

### 3.8 Validation of differential gene expression by qRT-PCR

To validate the accuracy of the transcriptome analysis, 9 DEGs were randomly selected for quantitative real-time qRT-PCR analysis for comparison of their expression levels among four comparisons (SE1, SE2, GE1 and GE2) (**Supplementary Figure S3**). The correlation between the RNA-Seq and qRT-PCR results was analyzed using Excel 2019, revealing a highly significant correlation (r = 0.7859) between the qRT-PCR and RNA-Seq data (p<0.01) (**Supplementary Figure S4**). This result indicates that the transcriptome analysis was accurate.

## 4 Discussion

Fruit setting represents the transition from the static state of the ovary development to the active state of fruit development. This process involves the pollination and fertilization of the ovule, followed by the growth of the ovary structure. However, some species have the ability to undergo parthenocarpy, allowing the ovary to set without pollination and fertilization. The understanding of parthenocarpy in tomato, strawberry, and cucumber have been elucidated by examining changes in fruit phenotype and ovary cell structure in the early stages of ovary development (Li et al., 2014; Kim et al., 2020; Zhou et al., 2021). Unpollinated fruit setting rate and fruit size can provide insights into the degree of parthenocarpy to some extent (Lietzow et al., 2016; Bermejo et al., 2018). In this study, by observing the fruit phenotypes at the early stage of ovary development in each treatment, it was found that the ovary began to exhibit significant growth on the 10th day after anthesis, with rapid growth observed on the 20th to 26th day after anthesis (**Figure 1A**). Notably, the emasculation G pomelo showed significantly larger compared to the emasculation S pomelo (**Figure 1A**, **Supplementary Figure S1**). Previous studies have shown that the number of cell layers and cell size of the ovary wall of Satsuma mandarin, which has strong parthenocarpic capacity, were significantly higher than that of Clementine Tangerine in the early stage of ovary development (Mesejo et al., 2016). According to our cytological observation, we found that the ovary wall thickness and cell layer number of G pomelo, 10 days after emasculation, were significantly higher than those of other treatments (including the emasculation treatment of S pomelo) (**Figure 2C**). This indicates that one of the reasons for the parthenocarpy of G pomelo might be its ability to maintain strong cell division after emasculation.

Auxin, as one of the key hormones, acts as the initial signal for pomelo cell development and subsequently stimulates the synthesis of cytokinin and gibberellin (**Figure 3**), which is consistent with previous reports (Yang et al., 1996). IAA, GA3 and ZT jointly promote fruit expansion and cell division (**Figure 2**). The harmonization of the levels of the three elements can better reflect the effects of various hormones by the ratio (T value) of abscisic acid content. In tomato, the homeostatic balance of auxin and gibberellin plays an important role in fruit setting and fruit development (He and Yamamuro, 2022; Ezura et al., 2023). Our results showed that the T value change trend of first increasing during development and then decreasing after 20 DAA in emasculated G pomelo exhibit similarities to those in pollinated S pomelo and G pomelo, but it was worth mentioning that the pollinated S pomelo and G pomelo decreased after 10 DAA (**Figure 3E**), indicating emasculation may have delayed fruit development. While the emasculated S pomelo did not show significant changes, which may indicate that the ovary did not develop obviously (**Figure 3E**). Therefore, the low fruit rate and weak parthenocarpic ability of unpollinated S pomelo may be due to the insufficient supply of growth-promoting hormones.

The first physiological fruit drop in citrus typically occurs 10 to 15 days after flower abscission, and it is primarily attributed to changes in carbohydrate metabolism and disruption of endogenous hormone balance. In this experiment, the T value of hybrid-pollinated and unpollinated treatments of S pomelo and G pomelo reached a low level at 26 days after flowering (**Figure 3E**), which may be closely related to the initial physiological fruit drop in citrus. Meanwhile, the T value of unpollinated G pomelo was higher than that of unpollinated S pomelo, corresponding to the higher fruit setting rate of unpollinated G pomelo one month after flowering (**Table 1**). This indicated that the endogenous hormone balance can affect the fruit drop. However, the T value of pollinated S pomelo was the lowest at 26 days after flowering, but its fruit setting rate was the highest (**Figure 3E**; **Table 1**). During this time, ZT and GA_3_ contents of pollinated S pomelo were significantly higher compared to other treatments (**Figures 3B, C**), suggesting that elevated levels of cytokinin and gibberellin accumulation could prevent physiological fruit drop in pomelo. Previous studies have shown that spraying 2,4-dichlorophenoxy acetic acid (2, 4-D) on citrus can effectively reduce physiological fruit drop (Kaur et al., 1997; Stander et al., 2014), and the underlying principle may involve enhancing endogenous gibberellin and cytokinin synthesis to prevent fruit drop (Cong et al., 2019), which is consistent with the findings of this experiment. However, the specific mechanism requires further investigation. In addition, studies have shown that citrus peel and pedicel are more effective in assessing the effects of endogenous hormones on pre-harvest fruit drop (Dong et al., 2020). Therefore, this experiment will contribute to a deeper understanding of the mechanism by which endogenous hormones affect parthenocarpy fruit setting or physiological fruit drop by distinguishing various fruit parts for endogenous hormone determination.

**Figure 8:**
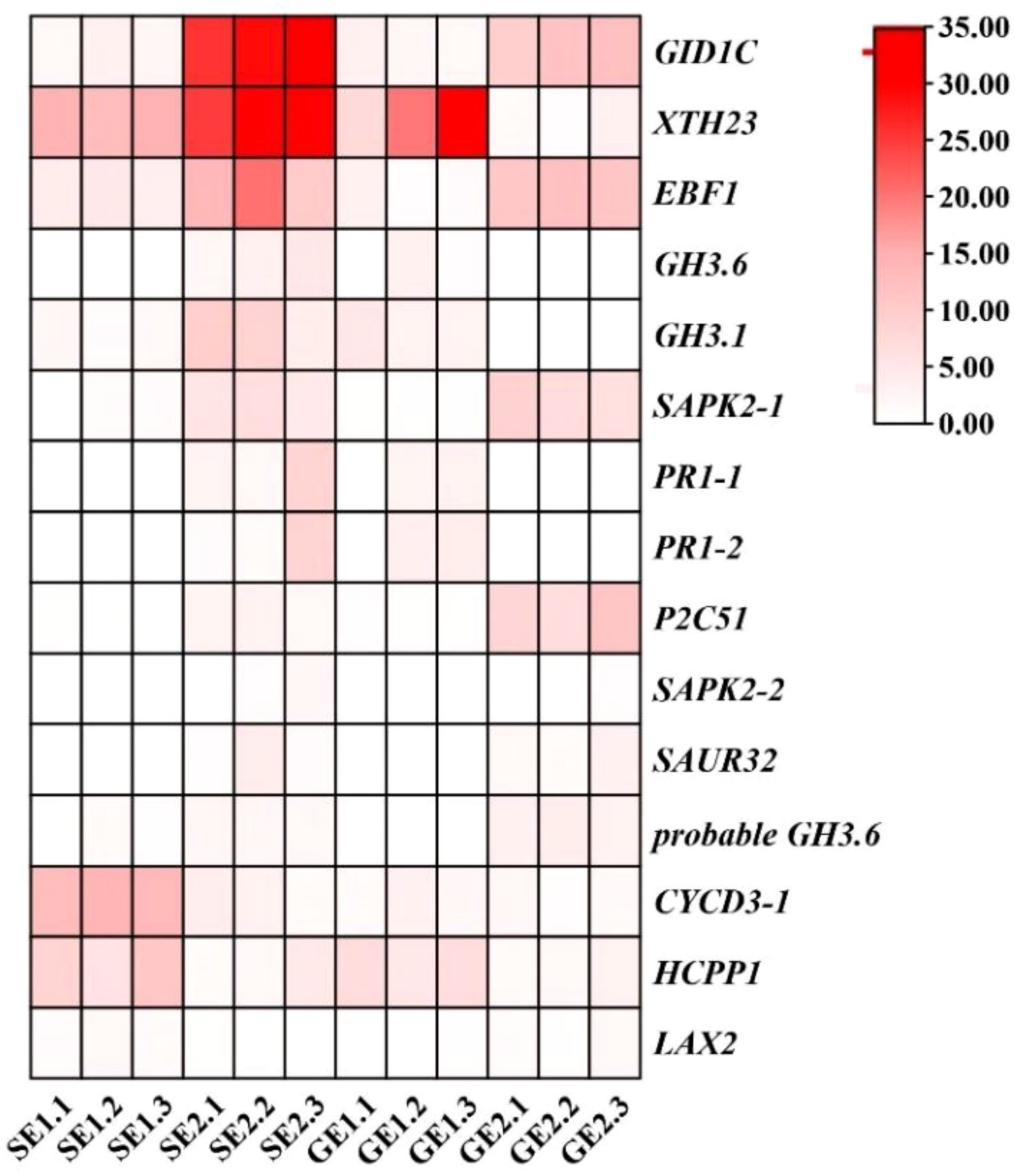
Transcriptome expression levels of DEGs in the plant hormones pathway of SE1 vs SE2 and GE1 vs GE2 group.

RNA-seq has been used in a variety of horticulture plants for rapid screening of parthenocarpy related candidate genes (Liu et al., 2017; Pomares-Viciana et al., 2019). However, this technology has not been applied to screen for effective parthenocarpy genes in citrus. In this study, the ovaries of S pomelo and G pomelo were used as materials at 10 d and 26 d after emasculation. Through pairwise comparisons of DEG analysis, it was found that the SE2 vs. GE2 comparison group exhibited the highest number of DEGs (4,635), while the SE1 vs. GE1 comparison group had the lowest number of DEGs (1,525) (**Figure 4A**). The functional analysis of DEGs confirmed that the parthenocarpic fruit exhibited specific transcripts and functions at 26 days after emasculation. In some horticultural crops, plant hormones are associated with parthenocarpy, where auxin, gibberellins, and cytokinins are the main players in initiating fruit set. The synergistic and antagonistic crosstalk between these hormones and others (such as ethylene, brassinosteroids, and melatonin) affects fruit set (Sharif et al., 2022). Consistent with this, this research showed that plant hormones may be involved in parthenocarpy in pomelo (**Figure 3**). The KEGG analysis revealed that plant hormone signal transduction pathway-related differentially expressed genes (DEGs) were highly enriched in SE1 vs. SE2 (**Supplementary Figure S5A**) and GE1 vs. GE2 (**Supplementary Figure S5B**) comparisons. Further analysis showed that the Venn diagram of plant hormone signal transduction pathway exhibited 15 common DEGs between SE1 vs. SE2 and GE1 vs. GE2 comparisons (**Supplementary Figure S5C**, **Figure 8**), and 6 shared DEGs between SE1 vs. GE1 and SE2 vs. GE2 comparisons (**Supplementary Figure S5D**, **Supplementary Table S4**). Among these 15 common DEGs, 5 genes displayed opposite transcriptional regulation trends in the former two comparisons, whereas the other 6 shared DEGs exhibited reversed expression trends between the latter two comparisons. These DEGs (*PR1-1, PR1-2, LAX2, GH3.1, GH3.6, XTH23, ERF1B*) were hypothesized to potentially regulate parthenocarpy in G pomelo. The qRT-PCR showed that the transcription levels of *CgERF1B*, *CgLAX2* and *CgGH3.6* showed a trend of first increasing and then decreasing during fruit development, while the transcription level of *CgGH3.1* showed a trend of decreasing in GE vs SE (**Figure 8**). The AUX1/LAX gene family is the key of auxin influx carrier, is mainly responsible for regulating auxin transport between cells (Staswick et al., 2005). *CgGH3.1* and *CgGH3.6* catalyze the synthesis of indole-3-acetic acid (IAA) -amino acid conjugates, providing a mechanism for plants to cope with the presence of excess auxin (Swarup and Bhosale, 2019). This suggests that the related-genes of auxin and ethylene may be jointly involved in parthenocarpy of pomelo, which is similar to the report of zucchini (*Cucurbita pepo L.*) (Pomares-Viciana et al., 2019).

**Figure 9:**
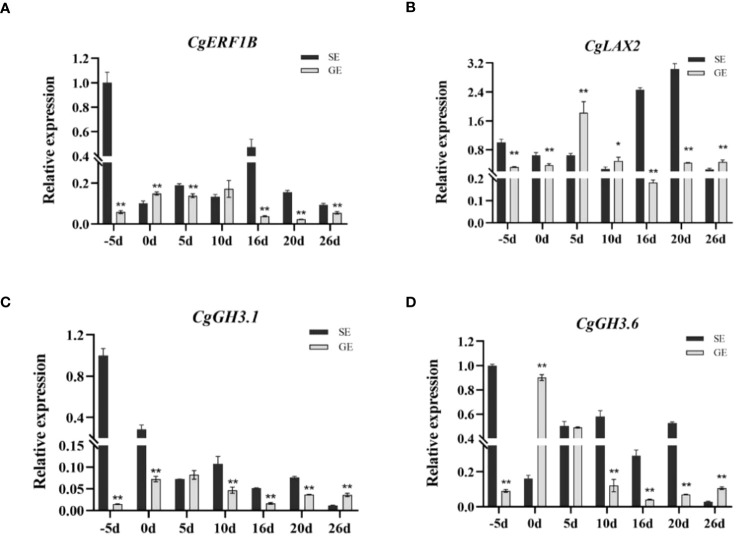
Verification of the expression of selected DEGs by qRT-PCR. The expression patterns of the genes CgERF1B **(A)**, CgLAX2 **(B)**, CgGH3.1 **(C)**, and CgGH3.6 **(D)**. Error bars indicate the standard deviation of three biological replicates. According to T test, the asterisk “*” indicates a statistically significant difference at the *p*<0.05 level, and the double asterisk “**” indicates a statistically significant difference at the *p*<0.01 level.

By comparing the encoding ability of TFs in each group, we identified the transcription factor gene families that exhibited higher numbers of more differential expression numbers in parthenocarpic pomelo cells. Among them, the *ERF*, *C2H2*, *bHLH*, *NAC* and *MYB* families showed significant representation (**Figure 7**). *ERF*, as an ethylene response element binding factor, belongs to a large class of transcription factors in plants. It plays essential roles in various biological and physiological processes throughout the life cycle of higher plants (Feng et al., 2020). For example, *ERF* gene family members are involved in the development and ripening of peach (Zhou et al., 2020) and durian (Khaksar and Sirikantaramas, 2021) fruits. The quantitative results showed that the expression of *CgERF1B* changes in the parthenocarpy and the development of the fruit (**Figure 9**). These results were similar to peach and durian, indicated that *CgERF1B* was involved in fruit development in pomelo parthenocarpy. C2H2 transcription factors are known to regulate unique processes in plant life (Takatsuji, 1998). Studies have shown that some C2H2 zinc finger proteins (ZFPs) participate in the development of plant pollen (Arrey-Salas et al., 2021) and are also involved in the development of parthenocarpy in banana (Sardos et al., 2016). This is similar to our results, ZFPs are involved in parthenogenesis in citrus. In addition, it has been reported in the literature, changes in transcript abundance of *bHLH* family members can regulate parthenocarpy in tomato and Arabidopsis (Ruiu, 2013). The down-regulation of NAC family members has been associated with promoting parthenocarpy in citrus, Arabidopsis, and litchi (Subbaraya et al., 2020), which was consistent with our findings. *GAMYB* is typically regulated by miRNA and plays a role in regulating plant reproductive development. It is involved in tomato ovule development and fruit setting (da Silva et al., 2017) and regulates parthenocarpy induced by gibberellin in grape (Wang et al., 2018). In conclusion, one or more transcription factors may play a role in pomelo parthenocarpy, which provides a basis for further analysis of molecular mechanisms of pomelo parthenocarpy.

The two pomelo cultivars possess distinct strengths and weaknesses. ‘Shatianyou’ (S pomelo), a prominent and renowned variety in China, is celebrated for its intensely sweet flavor and minimal acidity. It reaches maturity in mid to late November, and while it is somewhat challenging to peel, this trait contributes to its suitability for fruit stores and shipping. Its pulp is crisp and tender with rich sweet flavor (Deng, 2008) The results of this experiment showed that emasculation treatment can significantly reduce the number of plump seeds in both varieties (**Supplementary Table S2**), and significantly decrease the mature fruit size of S pomelo, while G pomelo’s fruit size remaining largely unaffected (**Supplementary Table S2**). Additionally, cross-pollination markedly boosts the levels of glucose, fructose, and sucrose in the mature fruit of S pomelo, while emasculation treatment significantly lowers these three sugars (**Table 2**). This could be a contributing factor to the necessity of artificial cross-pollination for S pomelo in production. ‘Guanximiyou’ (G pomelo), another popular variety, is favored for its well-balanced sweet and sour taste and seedless fruit. This preference aligns with the significantly higher citric acid content observed in G pomelo compared to S pomelo in this experiment (**Table 2**). G pomelo is known for its early maturity and high yield, with a ripening period from October to mid-November. However, its fruit storage quality is not as superior as S pomelo, due to the susceptibility of its juice vesicle to granulation (Deng, 2008).

This research employs transcriptome sequencing to identify candidate genes associated with parthenocarpy, with the intention of harnessing pertinent biotechnologies to develop S pomelos endowed with robust parthenocarpic ability (Conti et al., 2021). This could lead to the development of seedless new varieties with the excellent quality of S pomelo. The implications of this research extend beyond mere agricultural innovation; it holds profound significance for the cultivation of S pomelo by obviating the requirement for manual pollination, thereby curtailing labor expenses. In addition to its direct impact on pomelo production, this research also serves as a significant valuable reference for the broader investigation of parthenocarpy and the production of seedless fruits across fruit trees and horticultural crops. Furthermore, parthenocarpy is an important agricultural trait that induces the development of seedless fruits, which is an ideal characteristic for consumers (Sharif et al., 2022), the introduction of seedless S pomelo varieties promises enhanced economic returns.

## 5 Conclusions

In this study, we found that IAA, GA_3_ and ZT jointly promote fruit expansion and cell division in parthenocarpic pomelo. Furthermore, a first comparative transcriptome analysis was conducted between S pomelo and G pomelo at two critical stages after emasculation. A total of 5,792 DEGs were obtained, with 4,635 between SE2 and GE2, and 1,525 DEGs between SE1 and GE1. Hierarchical clustering, GO and KEGG analysis of the DEGs revealed that these DEGs were mainly involved in oxidoreductase activity, endomembrane system, organonitrogen compound catabolic process and carbon metabolism. *ERF*, *C2H2*, *bHLH*, *NAC* and *MYB* transcription factor families showed notable enrichment in both SE2 vs. GE2 and SE1 vs. SE2 comparisons. The analysis of key GO entries, KEGG pathways, and transcription factor gene families in the transcriptome showed that auxin and ethylene may be involved in parthenocarpy in pomelo. Our study elucidated the key genes and endogenous hormones associated with parthenocarpy in pomelo, providing a theoretical basis and foundation for further research on parthenocarpy and fruit set in pomelo. Further investigation into the molecular functions of these candidates may provide new insights into parthenocarpy in pomelo.

## Data availability statement

The data presented in the study are deposited in the NCBI repository, accession number PRJNA1135876.

## Author contributions

KZ: Data curation, Writing – original draft, Writing – review & editing, Investigation. YCZ: Data curation, Writing – original draft. SS: Writing – original draft. ZY: Writing – original draft. YZ: Writing – original draft. WN: Writing – original draft. XW: Writing – review & editing. HS: Writing – review & editing. JD: Writing – review & editing. SW: Writing – review & editing. DW: Writing – review & editing. QH: Writing – review & editing. QG: Writing – review & editing. GL: Writing – review & editing. SX: Writing – review & editing.

## Supplementary material

Supplementary Figure 1 Changes of fresh weight, vertical and transverse diameters of ovary in each treatment. Values are means ± standard errors (SEs) of ten ovaries. **(A)** Ovary fresh weight. **(B)** Ovary vertical diameters. **(C)** Ovary transverse diameters. DAA, days after anthesis. Different letters indicate significant differences at the 0.05 level.

Supplementary Figure 2 Correlation of sample gene expression level. SE1 and SE2: The S pomelo ovaries of emasculation treatment at 10 DAA and 26 DAA, respectively; GE1 and GE2: The G pomelo ovaries of emasculation treatment at 10 DAA and 26 DAA, respectively.

Supplementary Figure 3 Verification of the expression of selected DEGs by qRT-PCR. Error bars indicate the standard deviation of three biological replicates.

Supplementary Figure 4 DEGs of RNA-seq was verified by qRT-PCR.

Supplementary Figure 5 DEGs of the top 20 KEGG pathways with the smallest P value in the SE1 vs SE2 group **(A)** and GE1 vs GE2 group **(B)**. Venn diagram of SE1 vs SE2 and GE1 vs GE2 **(C)**, SE1 vs GE1 and SE2 vs GE2 **(D)**.

Supplementary Table 1 qRT-PCR primers for transcriptome verification.

Supplementary Table 2 Mature fruit size and seed number of each treatment. Different letters indicate significant differences at the 0.05 level according to Tukey’s Honest Significant Difference (HSD) test. According to T-test, the asterisk “*” indicates a statistically significant difference at the *p*< 0.05 level.

Supplementary Table 3 Quality analysis of the clean data.

Supplementary Table 4 Trends of DEGs in the plant hormones pathway of SE1 vs GE1 and SE2 vs GE2 group.
