# Supplementary material for: Comparative transcriptome analysis of two pomelo accessions with different parthenocarpic ability provides insight into the molecular mechanisms of parthenocarpy in pomelo (Citrus grandis)
Source: Front Plant Sci. 2024 Jul 29;15:1432166. doi: 10.3389/fpls.2024.1432166 (PMC11317442; doi:10.3389/fpls.2024.1432166)
Supplement: Supplementary Figure 1 — Changes of fresh weight, vertical and transverse diameters of ovary in each treatment. Values are means ± standard errors (SEs) of ten ovaries. (A) Ovary fresh weight. (B) Ovary vertical diameters. (C) Ovary transverse diameters. DAA, days after anthesis. Different letters indicate significant differences at the 0.05 level. [file Image_1.pdf]

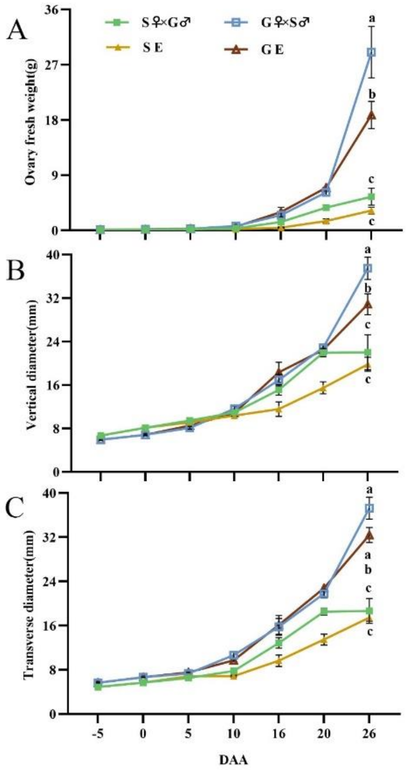

Fig.S1. Changes of fresh weight, vertical and transverse diameters of ovary in each treatment. Values are means ± standard errors (SEs) of ten ovaries. (A) Ovary fresh weight. (B) Ovary vertical diameters. (C) Ovary transverse diameters. DAA: days after anthesis. Different letters indicate significant differences at the 0.05 level.
