# Supplementary material for: Comparative transcriptome analysis of two pomelo accessions with different parthenocarpic ability provides insight into the molecular mechanisms of parthenocarpy in pomelo (Citrus grandis)
Source: Front Plant Sci. 2024 Jul 29;15:1432166. doi: 10.3389/fpls.2024.1432166 (PMC11317442; doi:10.3389/fpls.2024.1432166)
Supplement: Supplementary Figure 2 — Correlation of sample gene expression level. SE1 and SE2: The S pomelo ovaries of emasculation treatment at 10 DAA and 26 DAA, respectively; GE1 and GE2: The G pomelo ovaries of emasculation treatment at 10 DAA and 26 DAA, respectively. [file Image_2.pdf]

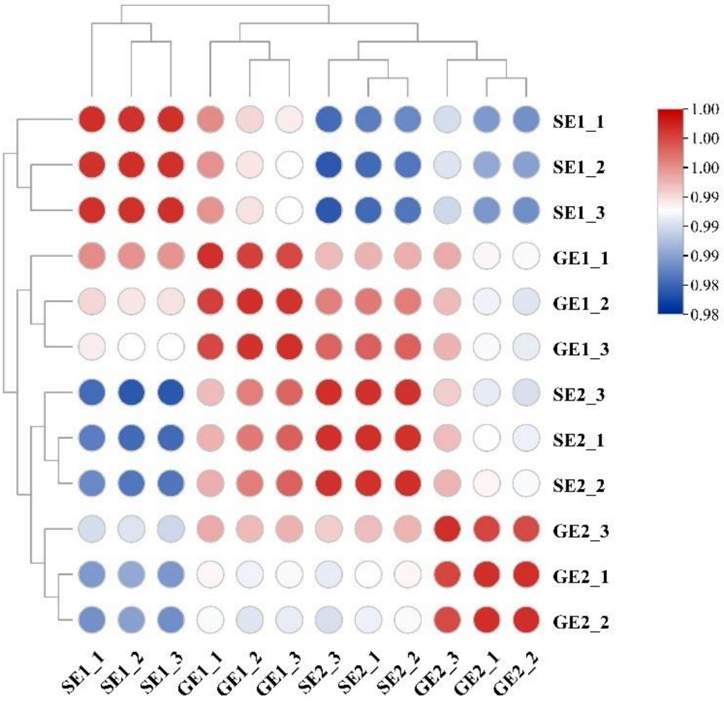


**Fig.S2.** Correlation of sample gene expression level. SE1 and SE2: The S pomelo ovaries of emasculation treatment at 10 DAA and 26 DAA, respectively; GE1 and GE2: The G pomelo ovaries of emasculation treatment at 10 DAA and 26 DAA, respectively.
