# Supplementary material for: Comparative transcriptome analysis of two pomelo accessions with different parthenocarpic ability provides insight into the molecular mechanisms of parthenocarpy in pomelo (Citrus grandis)
Source: Front Plant Sci. 2024 Jul 29;15:1432166. doi: 10.3389/fpls.2024.1432166 (PMC11317442; doi:10.3389/fpls.2024.1432166)
Supplement: Supplementary Figure 3 — Verification of the expression of selected DEGs by qRT-PCR. Error bars indicate the standard deviation of three biological replicates. [file Image_3.pdf]

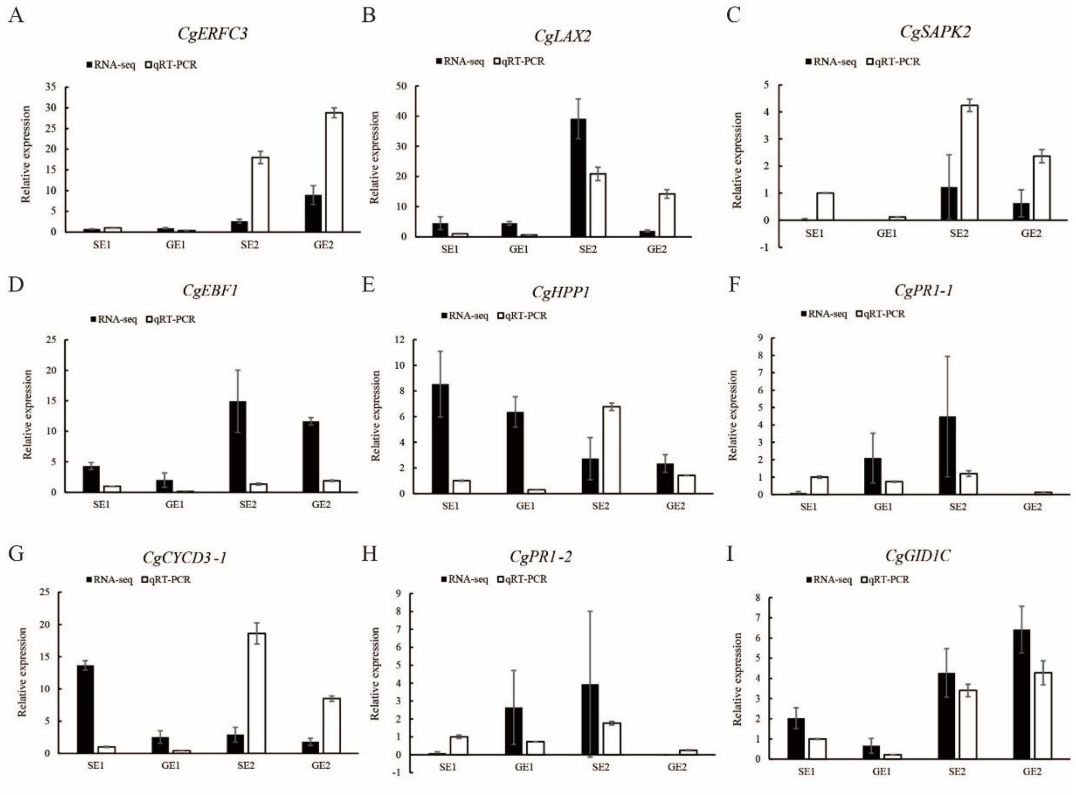

Fig.S3. Verification of the expression of selected DEGs by qRT-PCR. Error bars indicate the standard deviation of three biological replicates.
