# Supplementary figures and images for: Comparative transcriptome analysis of two pomelo accessions with different parthenocarpic ability provides insight into the molecular mechanisms of parthenocarpy in pomelo (Citrus grandis)
Source: Front Plant Sci. 2024 Jul 29;15:1432166. doi: 10.3389/fpls.2024.1432166 (PMC11317442; doi:10.3389/fpls.2024.1432166)

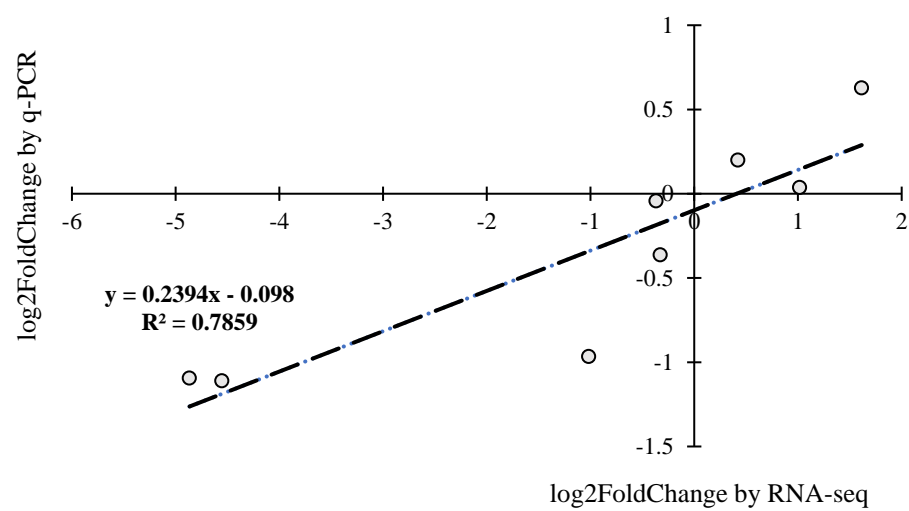

**Fig.S4.** DEGs of RNA-seq was verified by qRT-PCR.

Supplement: Supplementary Figure 4 — DEGs of RNA-seq was verified by qRT-PCR. [file Image_4.pdf]
