# Supplementary material for: Comparative transcriptome analysis of two pomelo accessions with different parthenocarpic ability provides insight into the molecular mechanisms of parthenocarpy in pomelo (Citrus grandis)
Source: Front Plant Sci. 2024 Jul 29;15:1432166. doi: 10.3389/fpls.2024.1432166 (PMC11317442; doi:10.3389/fpls.2024.1432166)
Supplement: Supplementary Figure 5 — DEGs of the top 20 KEGG pathways with the smallest P value in the SE1 vs SE2 group (A) and GE1 vs GE2 group (B). Venn diagram of SE1 vs SE2 and GE1 vs GE2 (C), SE1 vs GE1 and SE2 vs GE2 (D). [file Image_5.pdf]

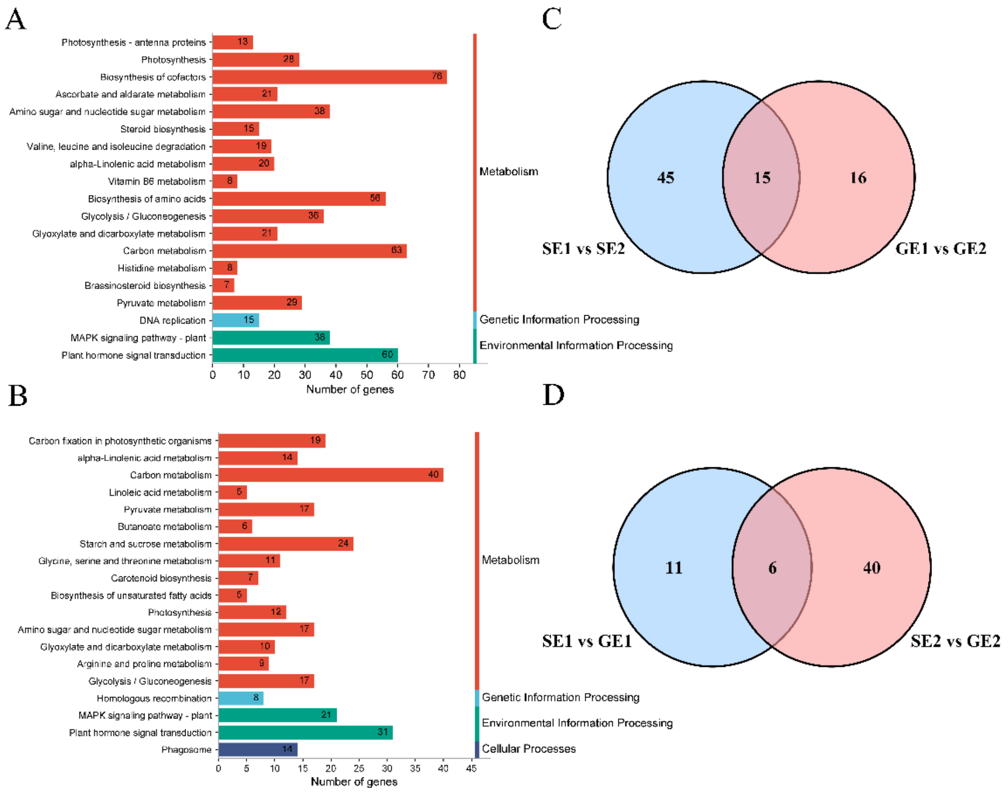

Fig.S5. DEGs of the top 20 KEGG pathways with the smallest P value in the SE1 vs SE2 group (A) and GE1 vs GE2 group (B). Venn diagram of SE1 vs SE2 and GE1 vs GE2 (C), SE1 vs GE1 and SE2 vs GE2 (D).
