# Supplementary material for: Comparative transcriptome analysis of two pomelo accessions with different parthenocarpic ability provides insight into the molecular mechanisms of parthenocarpy in pomelo (Citrus grandis)
Source: Front Plant Sci. 2024 Jul 29;15:1432166. doi: 10.3389/fpls.2024.1432166 (PMC11317442; doi:10.3389/fpls.2024.1432166)
Supplement: Supplementary Table 1 — qRT-PCR primers for transcriptome verification. [file Table_1.docx]

**Table S1** qRT-PCR primers for transcriptome verification

| Gene name | Primer（5’→3’） |
| --- | --- |
| *CgGID1C* | F：GGCACTTTCAATCGCCACTT |
|  | R：CTGCAAAGTAGGTTGGTGCC |
| *CgCYCD3-1* | F：CATCTCTGTCTAGGGCTCGC |
|  | R：TGTGTCTCCTCCACTTTGGC |
| *CgPR1-1* | F: TGCACAAGACTCACCCCAAG |
|  | R: GTTCTCGCCATACGGTCCTC |
| *CgPR1-2* | F：CATGCACAAGACTCACCCCA |
|  | R：TGCAGTCGCCTTTACGTTGA |
| *CgHPP1* | F：CACCGTAGCTTTAGACCAGCA |
|  | R：GGAGCTGCTACCCTTCAACT |
| *CgEBF1* | F：AACCCCAAGGAGTCAGGTCT |
|  | R：CTCCTCGCCTCCATCCAATC |
| *CgSAPK2* | F：GAAGGAGGGAGCTGGCAAAA |
|  | R：AATGGGCACACAAAATCGCC |
| *CgLAX2* | F：CTTTGGCATGGTGGGTCTGT |
|  | R：AGGACCCAGCAGTCCATCTA |
| *CgERFC3* | F：GTCACCGGAGTCTTTCCCTTT |
|  | R：TGATGTTGCTTCCGGTGTGG |
