# Supplementary material for: Comparative transcriptome analysis of two pomelo accessions with different parthenocarpic ability provides insight into the molecular mechanisms of parthenocarpy in pomelo (Citrus grandis)
Source: Front Plant Sci. 2024 Jul 29;15:1432166. doi: 10.3389/fpls.2024.1432166 (PMC11317442; doi:10.3389/fpls.2024.1432166)
Supplement: Supplementary Table 2 — Mature fruit size and seed number of each treatment. Different letters indicate significant differences at the 0.05 level according to Tukey’s Honest Significant Difference (HSD) test. According to T-test, the asterisk “*” indicates a statistically significant difference at the p< 0.05 level. [file Table_2.docx]

**Table S2** Mature fruit size and seed number of each treatment.

| Treatment | Fruit weight(g) | parthenocarpic fruit weight rate/% | Seed numbers | | |
| --- | --- | --- | --- | --- | --- |
|  |  |  | Normal seeds | | Abortive seeds |
| S natural | 761.5±13.12 c | / | 34±9.03 b | 115±10.31 ab | |
| S♀×G♂ | 916±28.57 c | / | 78±15.78 a | 77±16.58 b | |
| S E | 518±32.66 d | 57.17±5.29% | 0 c | 92±11.36 b | |
| G natural | 1385±53.71 b |  | 36±6.23 b | 112±8.94 ab | |
| G♀×S♂ | 1668±59.51 a |  | 59±14.78 ab | 88±15.49 b | |
| G E | 1572±170.39 ab | 94.75±10.33% * | 0 c | 135.4 ±17.49 a | |

Different letters indicate significant differences at the 0.05 level according to Tukey's Honest Significant Difference (HSD) test. According to T-test, the asterisk “*” indicates a statistically significant difference at the *p* < 0.05 level.
