# Supplementary material for: Comparative transcriptome analysis of two pomelo accessions with different parthenocarpic ability provides insight into the molecular mechanisms of parthenocarpy in pomelo (Citrus grandis)
Source: Front Plant Sci. 2024 Jul 29;15:1432166. doi: 10.3389/fpls.2024.1432166 (PMC11317442; doi:10.3389/fpls.2024.1432166)
Supplement: Supplementary Table 3 — Quality analysis of the clean data. [file Table_3.docx]

**Table S3** Quality analysis of the clean data

| Sample | Clean Reads Number | Base  Number | Q20(%) | Q30(%) | GC (%) | Mapping  rate (%) | Total  unigenes |
| --- | --- | --- | --- | --- | --- | --- | --- |
| SE1_1 | 61584766 | 9.09 G | 98.56 | 95.26 | 49.82 | 88.06 | 30113 |
| SE1_2 | 61755376 | 9.10 G | 98.37 | 94.97 | 49.58 | 86.67 |  |
| SE1_3 | 57847530 | 8.54 G | 98.45 | 95.03 | 49.625 | 87.42 |  |
| SE2_1 | 54422882 | 8.03 G | 98.30 | 94.86 | 50.09 | 83.33 |  |
| SE2_2 | 53956726 | 7.99 G | 98.10 | 94.51 | 49.66 | 82.42 |  |
| SE2_3 | 57068152 | 8.43 G | 98.19 | 94.60 | 49.71 | 82.09 |  |
| GE1_1 | 59630458 | 8.80 G | 98.46 | 95.00 | 50.735 | 90.53 |  |
| GE1_2 | 61083298 | 8.98 G | 98.47 | 94.95 | 50.815 | 87.76 |  |
| GE1_3 | 58343326 | 8.61 G | 98.41 | 95.01 | 50.825 | 89.27 |  |
| GE2_1 | 53417394 | 7.86 G | 98.49 | 94.94 | 50.615 | 93.66 |  |
| GE2_2 | 56258554 | 8.28 G | 98.54 | 95.09 | 50.43 | 93.79 |  |
| GE2_3 | 55955716 | 8.23 G | 98.58 | 95.18 | 50.01 | 92.89 |  |
