# Supplementary material for: Comparative transcriptome analysis of two pomelo accessions with different parthenocarpic ability provides insight into the molecular mechanisms of parthenocarpy in pomelo (Citrus grandis)
Source: Front Plant Sci. 2024 Jul 29;15:1432166. doi: 10.3389/fpls.2024.1432166 (PMC11317442; doi:10.3389/fpls.2024.1432166)
Supplement: Supplementary Table 4 — Trends of DEGs in the plant hormones pathway of SE1 vs GE1 and SE2 vs GE2 group. [file Table_4.docx]

**Table S4** Trends of DEGs in the plant hormones pathway of SE1 vs GE1 and SE2 vs GE2 group

| Gene | Name | SE1 vs GE1  Stat | SE2 vs GE2  Stat |
| --- | --- | --- | --- |
| XTH23 | probable xyloglucan endotransglucosylase/hydrolase protein 23 | up | down |
| ERF1B | ethylene-responsive transcription factor 1B | up | down |
| PR1-1 | pathogenesis-related protein 1-like | up | down |
| GH3.1 | probable indole-3-acetic acid-amido synthetase GH3.1 | up | down |
| GH3.6 | indole-3-acetic acid-amido synthetase GH3.6 | up | down |
| PR1-2 | pathogenesis-related protein 1-like | up | down |
